# Intradural extramedullary spinal choristoma: A case report

**DOI:** 10.1097/MD.0000000000036672

**Published:** 2023-12-15

**Authors:** XueRui Wu, Xue-Liang Cheng, Ming-Yang Kang, Rong-Peng Dong, Yang Qu, Jian-Wu Zhao

**Affiliations:** a Department of Orthopedics, The Second Hospital of Jilin University, Changchun, Jilin Province, China.

**Keywords:** choristoma, diagnosis, intraspinal tumor, magnetic resonance imaging, pathology, spine

## Abstract

**Rationale:**

A choristoma is a rare and benign neoplasm characterized by the presence of normal tissue in an anomalous anatomical location. In contrast, choristoma tend to occur in other body regions rather than within the spinal canal. Before our findings, only 4 cases of intraspinal choristoma had been recorded. Because its composition is complex and very rare, routine examinations, such as magnetic resonance imaging, are difficult to diagnose, and the possibility of its occurrence is often missed in clinical diagnosis. If there is no specificity in its components, such as in this case, even pathological examinations can only confirm the diagnosis as choristoma after eliminating other possibilities. Therefore, in clinical practice, when encountering patients with intraspinal tumors, it is essential to consider the possibility of choristoma. In this case, the choristoma lack of specific constituent composition sets it apart from previously reported intraspinal choristoma, significantly raising the diagnostic challenge, which offers valuable insights for clinical diagnosis.

**Patient concerns:**

A female patient aged 48 years was admitted to our medical center due to experiencing persistent lower back pain accompanied by radiating pain in both legs for 5 months. Based on the findings from the neurological physical examination and magnetic resonance imaging, the patient was diagnosed with an intradural space-occupying lesion located at the level of the first lumbar vertebral body. We performed an enhanced magnetic resonance neurography examination to further determine the positional relationship between the occupation and nerves in preparation for surgery. Postoperative pathological biopsy showed that the mass was an intraspinal choristoma.

**Diagnosis:**

Intradural extramedullary spinal choristoma.

**Intervention:**

Occupied lesion is removed surgically.

**Outcomes:**

After surgery, all symptoms were significantly relieved, and when the patient was discharged, all symptoms disappeared completely. There was no sign of recurrence after 1 year of follow-up.

**Lessons:**

Intraspinal choristomas are not specific and need to be diagnosed by pathologic examination. Early detection of and intervention for intraspinal tumors can mitigate nerve dysfunction.

## 1. Introduction

A choristoma is characterized by the presence of normal tissues or cells in an anomalous location.^[1]^ While this phenomenon is commonly observed in certain parts of the body, particularly in the tongue,^[2]^ its occurrence in the spinal canal is exceedingly uncommon. To date, only 4 cases of spinal choristoma have been recorded.^[3–6]^ This report presents a unique case of a choristoma situated in the lumbar spinal canal. The clinical manifestations, imaging findings, and pathological alterations of this unusual case are discussed in detail, providing novel insights distinct from those of previously reported cases.

## 2. Case report

### 2.1. Clinical summary

**Figure 1.**
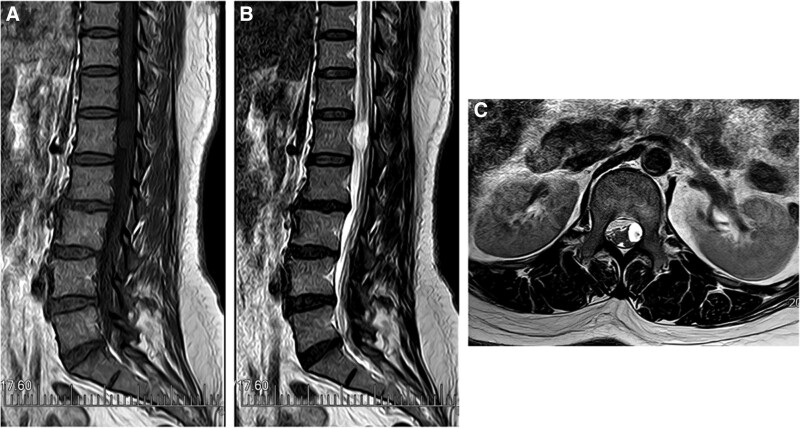
(A) Preoperatively sagittal T1-weighted lumbar spine MRI scan. (B) Preoperatively sagittal T2-weighted lumbar spine MRI scan. (C) Preoperatively axial T2-weighted lumbar spine MRI scan. MRI = magnetic resonance imaging.

**Figure 2.**
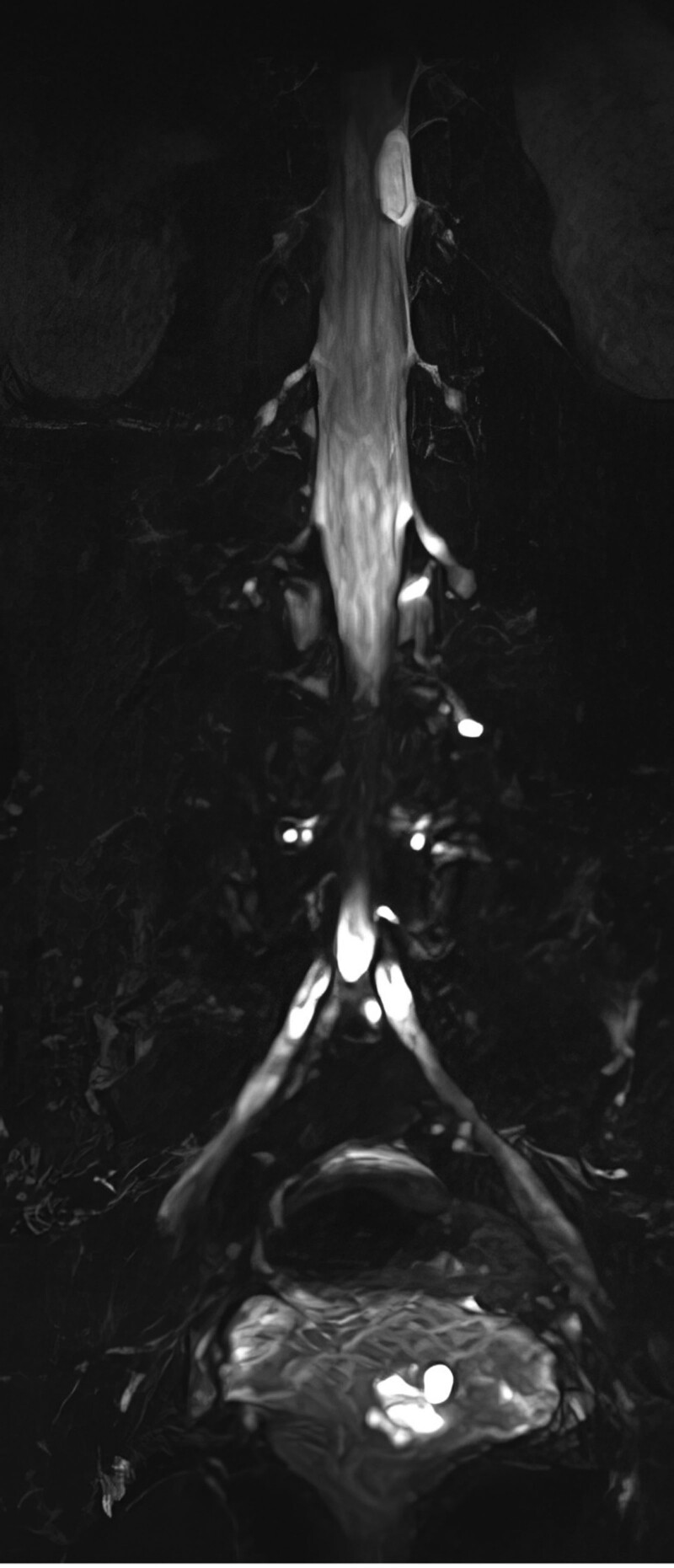
Preoperatively enhanced magnetic resonance neurography of the lumbosacral plexus.

**Figure 3.**
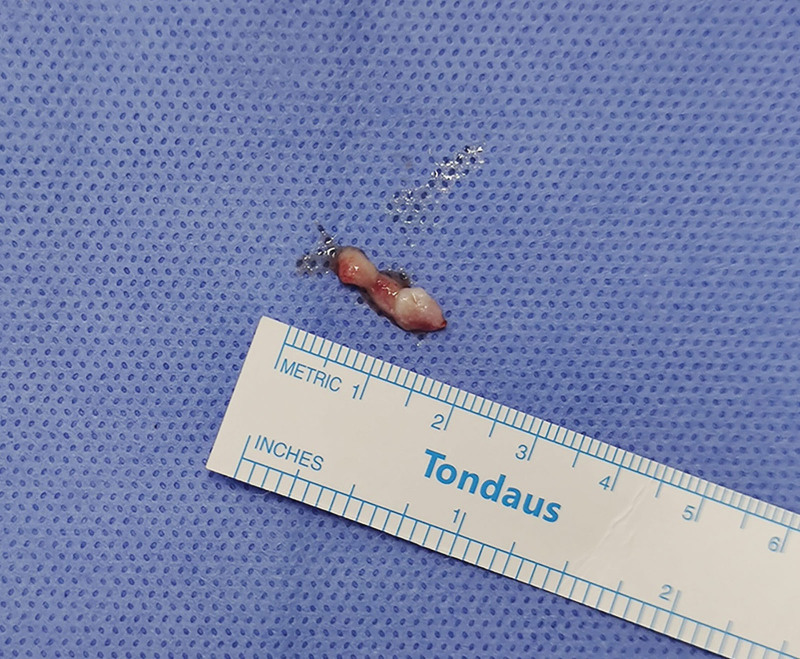
Intraoperative gross photograph of the choristoma.

**Figure 4.**
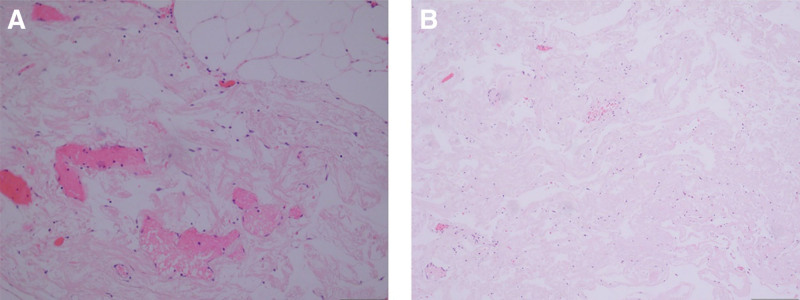
H&E staining image of choristoma (×100 magnification). (A) The blood vessels and adipose tissues were visualized in the choristoma. (B) Flattened epithelium could be observed in the choristoma.

A 48-year-old women was admitted to the department of orthopedics at The Second Hospital of Jilin University (Changchun, China), complaining of several symptoms, including severe lumbago, bilateral lower extremities pain, urinary, and defecation disorders. These symptoms had been bothering her for 5 months and began to worsen 10 days before her appointment. On physical examination, the patient is found to have tenderness and knocking pain over the lower lumbar area. Except for the weakness of the right extensor hallucis longus muscle, the muscle strength of the other lower limbs was normal. The patient’s bilateral straight leg raising test was negative, the Babinski sign was negative, and the bilateral knee tendon reflexes and Achilles tendon reflexes were normal. The lumbar magnetic resonance imaging (MRI) scan revealed the presence of an ovoid space-occupying lesion of size approximately 13 × 8 mm, exhibiting hypointense characteristics on T1-weighted images and hyperintense characteristics on T2-weighted images, located at the first lumbar vertebral body level (Fig. 1). An enhanced magnetic resonance neurography examination was performed on the lumbosacral plexus, which revealed abnormal enhancement in the extramedullary subdural region at the level of the first lumbar vertebral body. However, no significant abnormalities were observed in the bilateral lumbar nerves and the adjacent left first lumbar nerve (Fig. 2). Imaging shows mass lesions compressing the spinal cord, and the results are broadly consistent with the patient’s presenting symptoms and physical examination. Based on clinical evaluations, the diagnosed condition was an intraspinal space-occupying lesion situated at the L1 level, with a potential differential diagnosis of a lipoma or teratoma. The surgical procedure was performed while utilizing neurophysiological monitoring. The intraspinal tumor that was excised exhibited a grayish appearance, possessed a substantial vascularization, and demonstrated adherence to the adjacent nerve root (Fig. 3). The tumor was removed and the pathological examination indicated the presence of fibrous adipose tissue, with dilated, congested blood vessels seen internally, covered with flattened epithelium (Fig. 4). The immunohistochemical staining results were as follows: S-100(−), GFAP(−), Ki67(positive rate < 1%), CD34 (blood vessels positive), D2-40(+), CK(AE1/AE3)(−), EMA(−), CD31(−), SOX10(−), SMA(−). The final diagnosis was intraspinal choristoma at the L1 level. The patient showed satisfactory postoperative recovery, with significant relief from lower back pain and bilateral lower extremity pain, and urinary and bowel function returned to normal. Over a period of 1 year of follow-up, all clinical symptoms disappear completely.

## 3. Discussion

Choristomas are lesions consisting of normal cells or tissue that occur in an abnormal location.^[7]^ Since a choristoma is rarely found in the spinal cord, it is not usually suspected before surgery or pathologic examination. The differential diagnoses for spinal choristomas are hamartoma, teratoma, and lipoma. Typically, only benign lesions comprising disorganized mature tissue that is typically found in the affected area should be classified as pure hamartomas. Hence, spinal hamartomas primarily consist of well-differentiated, mature tissue derived from both ectodermal and mesodermal layers. Much like spinal choristomas, spinal hamartomas do not harbor the potential for malignant transformation. Accordingly, our study excluded hamartomas. Teratomas are neoplasms comprised of differentiated cell types originating from all 3 germ layers (ectoderm, endoderm, and mesoderm).^[8]^ These tumors can manifest on computed tomography scans with heterogeneous densities or calcifications.^[9]^ MRI is helpful for diagnosing teratomas, which exhibit a mixture of high and low intensity, thereby indicating heterogeneity in the tissues.^[10]^ By integrating both pathological and MRI findings, we were able to exclude teratomas as a possible diagnosis. Intradural spinal lipomas occur mainly in the thoracic and cervical vertebrae, which generally show hyperintense T1 and T2 signals. Lipomas of the lumbosacral region, histologically, are made up of mature adipose tissue arranged in lobules and accompanied by loose connective tissue.^[11]^ Therefore, lipomas were excluded from the study.

It is common for choristomas to grow slowly and to be asymptomatic, with dysfunction only occurring as they become larger. Using the keywords “choristomas,” “spinal choristomas,” and “intradural choristomas,” we conducted searches in the WoS and PubMed databases. We then selectively filtered articles based on their relevance to the topic “intradural extramedullary spinal choristom” and ultimately identified 4 articles that met our inclusion criteria. Kurman et al^[3]^ reported the first case of a choristoma originating from the lumbosacral region. Chen et al^[4]^ described a breast-related spinal choristoma masquerading as a lipomyelomeningocele of the lumbar spine. Pathological examination indicated that the glandular elements, encased by epithelial and myoepithelial cells within the spinal mass, bore resemblance to breast tissue. Chang et al^[5]^ presented a case of a lumbosacral choristoma derived from striated muscle. Throughout the pathological examination, the tumor was found to comprise both striated muscle and glandular tissue. Yang et al^[6]^ reported a case of intraspinal choristoma combined with spinal cord tethering syndrome in the lumbar region. In this report, we described a case of intradural extramedullary spinal choristoma at the lumbar segment, which was composed of fibrous adipose tissue and blood vessels and covered with flattened epithelium. Histology does not have specific components, such as striated muscle or glandular tissue, and there are no other comorbidities, which greatly increases the difficulty of diagnosis. This type of lesion is extremely rare and has not been previously reported.

## 4. Conclusion

Intraspinal choristomas exhibit a benign clinical course and histopathological features. Successful surgical excision and recovery are highly feasible if these lesions are detected and managed in a timely manner. However, preoperative diagnosis of intraspinal choristomas poses a significant challenge. Surgical specimens are typically necessary to confirm the diagnosis.

## Author contributions

**Conceptualization:** XueRui Wu, Ming-Yang Kang, Rong-Peng Dong, Yang Qu, Jian-Wu Zhao.

**Funding acquisition:** Yang Qu.

**Writing – original draft:** XueRui Wu, Xue-Liang Cheng.

**Writing – review & editing:** Yang Qu, Jian-Wu Zhao.
